# Comparison of Functional, Structural, and Microvascular Features in Different Stages of Idiopathic Epiretinal Membrane

**DOI:** 10.3390/jcm13113188

**Published:** 2024-05-29

**Authors:** Paola Marolo, Enrico Borrelli, Paolo Caselgrandi, Guglielmo Parisi, Francesco Gelormini, Federico Ricardi, Luca Ceroni, Matteo Fallico, Mario Damiano Toro, Luca Ventre, Michele Reibaldi

**Affiliations:** 1 Department of Ophthalmology, University of Turin, 10126 Turin, Italy; borrelli.enrico@yahoo.com (E.B.); caselgrandi.paolo@gmail.com (P.C.); guglielmoparisi@gmail.com (G.P.); francesco.gelormini@hotmail.it (F.G.); federico.ricardi@gmail.com (F.R.); michele.reibaldi@unito.it (M.R.); 2 Department of Psychology, University of Turin, 10124 Turin, Italy; cashlucas85@gmail.com; 3 Department of Ophthalmology, University of Catania, 95123 Catania, Italy; matteofallico@hotmail.com; 4 Department of General Ophthalmology, Medical University of Lublin, 20079 Lublin, Poland; mariodamiano.toro@unina.it; 5 Eye Clinic, Public Health Department, Federico II University, 80131 Naples, Italy; 6 Department of Ophthalmology, Beauregard Hospital, 11100 Aosta, Italy; lucaventre@yahoo.it

**Keywords:** epiretinal membrane, visual acuity, near vision, optical coherence tomography, macular pathology, surgical timing

## Abstract

**Background:** Idiopathic epiretinal membrane (ERM) often leads to visual symptoms such as metamorphopsia and decreased central vision. This study aimed to evaluate functional, structural, and microvascular characteristics in patients with different stages of idiopathic ERM who were candidates for surgery, with a focus on identifying potential indicators for surgical timing. **Methods:** A retrospective cohort study was conducted on consecutive patients with unilateral idiopathic ERM who were candidates for surgery. Patients underwent comprehensive ophthalmological assessments, including OCT grading, reading performance evaluation, and OCT angiography. Data analysis included comparisons between different ERM stages for functional, structural, and microvascular parameters. **Results:** A total of 44 eyes were included, classified into four ERM stages according to the Govetto grading system. Functional parameters, including distance and near visual acuity, worsened significantly with higher ERM stages, particularly in the transition from Stage 3 to Stage 4. Structural assessments revealed significant increases in central macular thickness (CMT) from Stage 3 to Stage 4. No significant differences were observed in microvascular features across different ERM stages. **Conclusions:** This study highlights the significant functional and anatomical impact of OCT staging in idiopathic ERM, particularly during the transition from Stage 3 to Stage 4, characterized by notable reductions in visual acuity and increases in CMT. These findings underscore the importance of considering both functional and structural parameters in surgical decision-making for ERM management. However, further research with larger cohorts is needed to confirm these observations and inform clinical practice.

## 1. Introduction

Idiopathic epiretinal membrane (ERM) is one of the most common macular pathologies in elderly people, with its prevalence increasing with age [1]. Retinal traction caused by fibrocellular proliferation and extracellular matrix deposition over the internal limiting membrane (ILM) is mostly asymptomatic in the initial stages; it remains stable in the vast majority of eyes, but it can progress with time in about 30% of cases, leading to structural alterations and visual symptoms such as metamorphopsia, visual blurring and decreased central vision [2,3].

Currently, structural optical coherence tomography (OCT) provides an objective method for the diagnosis of ERMs, and the widely adopted Govetto grading system classifies idiopathic ERMs into four stages of severity based on the OCT appearance of foveal depression and retinal layers [4]. In recent years, there has been a notable increase in OCT studies examining macular anatomical changes that can adversely affect visual function. These studies have played an important role in characterizing most retinal diseases of medical and surgical interest. Among the retinal pathologies of surgical interest, extensive evidence has been gathered on ERMs. This evidence has focused on aspects such as alterations in retinal anatomy, including the disruption of the ellipsoid zone (EZ) and photoreceptor outer segments [5,6,7]. Photoreceptor disruption detected using OCT has been identified as a predictor of poor visual recovery after ERM surgery [5]; moreover, vision loss and symptoms such as metamorphopsia have been shown to be significantly associated with the thickness of inner retinal layers [8,9,10]. However, in order to fully understand the severity of the pathology and to define indications for surgery, the patient’s visual symptoms and discomfort during daily activities should also be considered [11]. Furthermore, recent non-invasive methods of analyzing microvascular changes using optical coherence tomography angiography (OCTA) have proven useful for objectifying the progression of epiretinal membranes [12,13,14].

It is understood that a greater ERM OCT stage corresponds to reduced visual function and more visual symptoms, but few studies have investigated functional changes in the transition from one stage to another.

To date, distance best-corrected visual acuity (BCVA) measurement remains the gold standard for evaluating changes in visual function. However, visual changes in the transition between different stages can be varied and also cause metamorphopsia and a reduction in near vision [15]. Near vision is involved in many daily activities, and its impairment can have a significant impact on the patient’s quality of life (QoL); thus, BCVA examination alone may be insufficient to assess the discomfort imposed by the disease on the patient’s daily life.

The purpose of this study was to evaluate functional, structural, and microvascular characteristics in different stages of epiretinal membrane candidates for surgery and to understand whether there may be additional elements useful for the choice of surgical timing.

## 2. Materials and Methods

We performed a retrospective cohort study on consecutive patients affected by unilateral idiopathic ERM who were candidates for surgery at “Città della Salute e della Scienza” Hospital, University of Turin, Italy, and underwent a baseline examination between March 2023 and October 2023. The study protocol complied with the tenets of the Declaration of Helsinki and was reviewed and approved by the Institutional ethics committees. All patients signed a written informed consent form, agreeing to participate.

Idiopathic ERMs were classified into four different stages according to the Govetto staging scheme [4]. Briefly, Stage 1 is characterized by the presence of a recognizable foveal pit, well-defined retinal layers, and minimal morphological or anatomical disruption. Progressing to Stage 2, increased tangential traction from the epiretinal membrane (ERM) results in the loss of the foveal depression and stretching of the outer nuclear layer, while maintaining well-defined retinal layers. In Stage 3, persistent traction leads to the formation of an ectopic inner foveal layer (EIFL) and significant thickening with loss of the foveal pit, yet the retinal layers remain distinguishable. Lastly, Stage 4 is identified by indistinct boundaries between retinal layers and the EIFL.

Eyes with an ERM stage ≥ 2, BCVA ≥ 0.1 LogMAR, reported metamorphopsia, and documented progression of the membrane were candidates for surgery and included in the study. Exclusion criteria were: (1) ERM secondary to other vitreoretinal diseases; (2) ocular conditions other than ERM that may affect BCVA, including significant media opacity due to cataracts graded more than N03 or NC3 according to the Lens Opacity Classification System [16], other macular disorders such as age-related macular degeneration, and optic nerve diseases such as glaucoma; (3) previous ocular surgery except for uncomplicated cataract surgery; (4) axial length ≥26 mm or a spherical equivalent value ≤ −6 D; and (5) the presence of image artifacts during OCT or OCTA.

All patients underwent a complete ophthalmologic assessment, including slit lamp biomicroscopy and dilated fundus examination. Demographics and clinical characteristics including age, gender, lens status (pseudophakic or phakic), and axial length were collected.

The main objective of the study was to compare functional, structural, and microvascular features between three different stages of ERM candidates for surgery.

### 2.1. Reading Performance Assessment

Reading performance was assessed monocularly using the Italian version of Radner reading charts [17]. One eye for each patient was included. Radner charts include 41 sentences of standardized grammatical construction and lexical and syntactical complexity. Print sizes progress geometrically from 0.25 m to 6.3 m.

Before starting the test, a lensometer was used to obtain the refraction measure in patients wearing spectacles. Otherwise, patients underwent either retinoscopy or auto-refractometry to measure the BCVA. The background illumination, room setting, and reading position were the same during the test for each evaluation. Furthermore, chart luminance was 120 cd/m^2^, while the reading distance was set at 40 cm. Patients were asked to read the sentences on the Radner reading charts as fast as possible during the examination. Subjects were also asked to keep reading until they were not able to continue, even after the occurrence of mistakes (i.e., mispronunciations or unread syllables) that the examiner promptly tracked. The latter also recorded the time taken to read sentences. After the completion of the examination, the following metrics were obtained: (i) reading acuity was defined as the logarithm of the reading acuity determination (LogRAD) at the smallest sentence (print size) a patient is able to read in no more than 30 s (range, −0.2 to 1.3 logRAD) and (ii) the maximum reading speed (max RS) was defined as the fastest time achieved across the varying print sizes and is calculated in words per minute (wpm).

### 2.2. OCT Grading and Structural Assessment

All iERM eyes were evaluated and graded into 4 stages [4] using the Heidelberg Spectralis HRA + OCT (Heidelberg Engineering, Heidelberg, Germany) spectral domain (SD) OCT with a volumetric scan protocol composed of 19 horizontal B-scans, 24× averaging for each B-scan, covering approximately a 5.5 × 4.5-mm area (20° × 15°) centered on the fovea. To be included, the scans were also required to have a minimum signal strength index of 25, as advised by the manufacturer [18].

The retinal thickness map analysis protocol was selected to analyze numeric averages of the measurements for central macular thickness (CMT). Furthermore, the morphology of the fovea was qualitatively evaluated in each patient. Tractional central foveal bouquet abnormalities were identified and classified as the presence of a cotton ball sign, a foveolar detachment, or an acquired vitelliform lesion, as previously described [19]. Also, the presence of an EZ disruption and tractional cysts were noticed. EZ disruption was defined as discontinuity of the ellipsoid band in the foveal region [4]. In contrast, the presence of an area of EZ attenuation located at the site of a round or diffuse hyperreflective area between the EZ and the cone outer segment tip line at the center of the fovea was identified as the cotton ball sign [20]. Tractional cysts were identified as hyporeflective intraretinal cystoid spaces between the outer nuclear and outer plexiform layers and in the inner nuclear layer of presumably tractional origin [4,21].

### 2.3. Microvascular Assessment

OCTA images were obtained using the Zeiss Cirrus HD-OCT 5000 Angioplex (sw version 10.0, Carl Zeiss Meditec, Inc., Dublin, OH, USA). OCTA involved scanning a 3 × 3 mm^2^ and 6 × 6 mm^2^ foveal-centered area. FastTrac retinal tracking technology (Ophthalmology Web, San Francisco, CA, USA) was used to minimize motion artifacts. A signal strength of 6 of 10 or greater was accepted. All scans were analyzed using en-face OCTA images generated automatically using Angioplex software.

The FAZ parameters were evaluated on the 3 × 3 mm^2^ scan and measured in the slab corresponding to the superficial plexus using automated Angioplex software. The entire en-face microvasculature was evaluated in the 6 × 6 mm^2^ area. Superficial retinal capillary plexus (SCP) was automatically segmented between the internal limiting membrane (ILM) and the outer boundary of the inner plexiform layer (IPL). Macular regions were automatically segmented into sectors defined by the Early Treatment of Diabetic Retinopathy Study (ETDRS). Specific parameters of interest were FAZ area and perimeter; vessel density and perfusion of the SCP (central, inner, and total length of perfused vasculature per unit area in the region of measurement).

All OCT and OCTA images were reviewed for eligibility, assessed for the presence of artifacts, and graded by two independent reviewers (F.G. and F.R.). Subsequently, the two readers met to assess their agreement, and disagreements were resolved by further discussion and open adjudication to yield a single result for each case. For those cases in which the two readers were unable to reach an agreement on a single consensus result, the final decision was made by a third senior retinal expert (M.R.). In detail, OCTA motion artifacts included blink lines, displacement, stretch artifacts, quilting, and vessel doubling as previously explained [22]. Also, the accuracy of segmentation was evaluated for all reference planes in all OCT scans. The choriocapillaris directly beneath superficial retinal vessels was excluded from the analysis to eliminate potentially confounding shadow or projection artifacts.

### 2.4. Statistical Analysis

Snellen visual acuity was determined and converted into the logarithm of the minimum angle of resolution (LogMAR) for statistical analysis. The mean and standard deviation (SD) were computed for continuous variables, while frequency and percentage were calculated for qualitative variables. The normal distribution of continuous variables was evaluated by using the Shapiro–Wilk test. The Kruskal–Wallis test and the Pearson chi-squared test were used to explore differences between the three stages for continuous and categorical variables, respectively. If significant, multiple comparisons between Stages 2 and 3, Stages 3 and 4, and Stages 4 and 2 were fitted by using the Mann–Whitney U test.

Variables that may potentially influence distance visual function were evaluated in univariate analyses with BCVA set as a dependent variable, using chi-squared or Fisher’s exact tests for categorical variables; for quantitative variables, since the distributions were not normal (Shapiro–Wilk test), the Mann–Whitney test was used. An odds ratio (OR) with a 95% confidence interval (CI) was also calculated; for continuous variables, Exp(B) from binary logistic regression was used. Logistic regression analysis was performed to test for independence between each variable and visual function. Variables that were significant at the *p* < 0.2 level in the univariate analysis were included in the logistic regression.

Statistical analysis was performed using Statistical Package for Social Sciences (28.0.1.0 version IBM SPSS Statistic Inc., Chicago, IL, USA). A *p*-value ≤ 0.05 was considered significant.

## 3. Results

A total of 44 eyes (44 patients) with ERM from 59 to 82 years of age (mean age: 70.44 ± 8.88 years) were enrolled, including 19 women and 25 men. Approximately 43.2% (19 eyes) of affected eyes were pseudophakic, and none of the phakic eyes suffered from cataracts affecting visual function. The mean axial length was 24.29 ± 1.25 mm.

**Figure 1:**
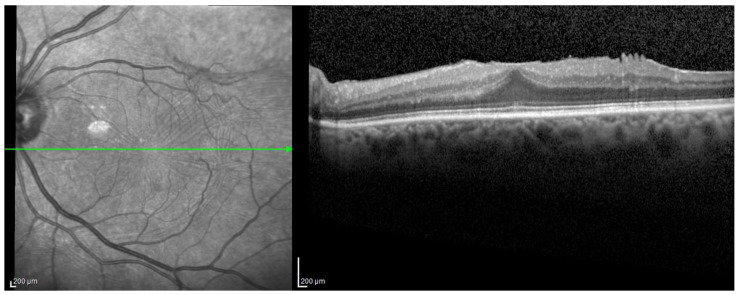
Stage 2 epiretinal membrane. The foveal depression is absent and retinal layers are well-defined. The outer nuclear layer shows signs of stretching, while the inner nuclear and ganglion cell layers seem displaced, and there is no continuous layer of retinal tissue over the outer nuclear layer.

**Figure 2:**
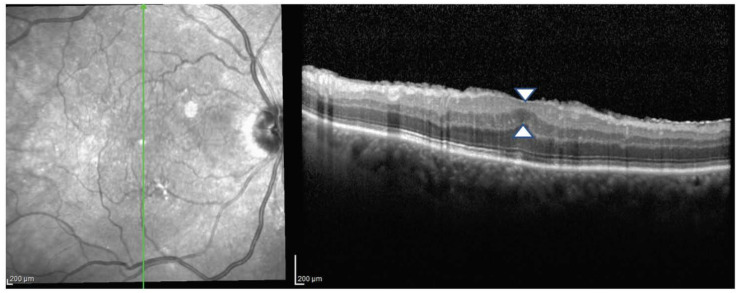
Stage 3 epiretinal membrane. The foveal depression is absent and retinal layers are well-defined. White arrows show the ectopic inner foveal layers above the outer nuclear layer, which appear continuous and span the entire foveal area.

**Figure 3:**
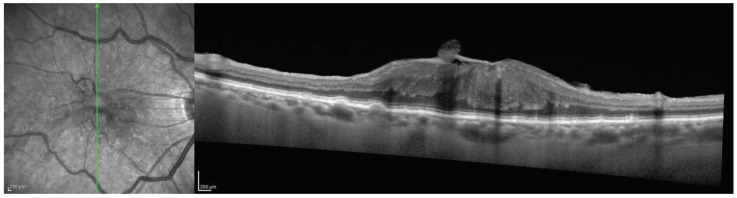
Stage 4 epiretinal membrane. The foveal depression is lost, and retinal layers are disrupted with poorly defined borders. The epiretinal membrane is thick, with evident traction affecting the underlying retina and the presence of ectopic inner foveal layers. Alterations are present in both the external limiting membrane and the ellipsoid zone.

All included eyes were candidates for surgery and were classified according to the Govetto staging system of ERMs [4]. None of the eyes (0%) were classified as Stage 1, ten eyes (22.7%) were classified as Stage 2 (Figure 1), twenty-four eyes (54.4%) were classified as Stage 3 (Figure 2), and ten eyes (22.7%) were classified as Stage 4 (Figure 3). No difference was found between the three stages when comparing the demographics and clinical characteristics of included patients (Table 1).

### 3.1. Comparison between Functional Parameters in Different Stages of ERM

As shown in Table 2, ERM eyes had an overall worse LogMAR BCVA in accordance with the ERM stage (Kruskal–Wallis, *p* < 0.01). A statistically significant difference was found between Stages 3 and 4 (Mann–Whitney, *p* = 0.012) and between Stages 2 and 4 (Mann–Whitney, *p* = 0.003). Similarly, overall worse LogRAD reading acuity and maximum reading speed were found in eyes with a higher stage of ERM (Kruskal–Wallis, *p* < 0.01). A statistically significant difference was found between Stages 3 and 4 (Mann–Whitney, *p* = 0.002 and *p* = 0.013, respectively), and between Stages 2 and 4 (Mann–Whitney, *p* = 0.013 and *p* = 0.003, respectively).

Abnormal Amsler grid test findings were obtained with an increasing trend according to the ERM stage (60.0% Stage 2, 75% Stage 3, and 100% Stage 4; Pearson chi-squared test, *p* = 0.095).

### 3.2. Comparison between Structural Parameters in Different Stages of ERM

Table 3 shows a comparison between structural parameters obtained using OCT in different stages of ERM. Central macular thickness varied significantly among the three stages of ERM, with an increased thickness in higher stages of ERM (Kruskal–Wallis, *p* = 0.004). CMT was thicker in Stage 4 compared with Stage 3 (Mann–Whitney, *p* = 0.006) and in Stage 4 compared with Stage 2 (Mann–Whitney, *p* = 0.003).

Tractional abnormalities of the central bouquet were diagnosed in 11 out of 44 eyes (25.0%). The most frequent subtype was the cotton ball sign (5 out of 11 eyes or 45.5%), followed by the acquired vitelliform lesion (4 out of 11 eyes or 36.4%) and foveolar detachment (2 out of 11 eyes or 18.2%). In Stage 2 ERM, the most common central bouquet abnormality was the cotton ball sign subtype (3 out of 10 eyes or 30%), and in Stage 3 ERM, it was the acquired vitelliform lesion (3 out of 24 eyes or 12.5%).

There was not a statistically significant difference between EZ disruption rates in different stages of ERM (Pearson chi-squared test *p* = 0.292); however, an increasing trend was noted in higher ERM stages. Also, a similar rate of tractional cysts was observed (Pearson chi-squared test *p* = 0.548).

None of the eyes showed ILM detachment or evidence of vitreomacular traction and/or adhesion.

### 3.3. Comparison between Microvascular Features in Different Stages of ERM

OCT angiography results are reported in Table 4. Mean values of the foveal avascular zone (FAZ) area and perimeter showed a decreasing trend in eyes with a higher ERM stage; however, our analysis showed no statistically significant difference between the three stages of ERM (Kruskal–Wallis test, *p* = 0.885 and *p* = 0.915, respectively).

Considering the superficial capillary plexus, we did not observe significant changes between vessel density and perfusion in different ERM stages (Kruskal–Wallis test, *p* > 0.05); nevertheless, an increasing trend with higher-stage ERMs was observed for central, inner, and total vessel density, as well as for central, inner, and total perfusion.

### 3.4. Multivariate Logistic Regression

Multivariate logistic regression was conducted on variables that were significant upon univariate analysis and showed that the following factors were significantly associated with BCVA: reading acuity (*p* < 0.01) and maximum reading speed (*p* < 0.01) (Table 5).

## 4. Discussion

In this study, we investigated functional, structural, and microvascular features in different stages of idiopathic epiretinal membrane candidates for surgery. We found the worsening of both best-corrected distance and near visual acuity and an increase in central macular thickness in the transition between Stages 3 and 4, while we did not observe any difference between Stages 2 and 3. The remaining structural and OCT angiography parameters, instead, remained stable throughout the three investigated stages.

Patients who are visually impaired due to macular disease often complain of a reading disorder as their first symptom [23]; therefore, we evaluated, in addition to BCVA, near vision as well through the functional parameters of reading acuity and maximum reading speed. It has been proven that many daily living tasks are related to reading ability with a consequent notable impact on the patient’s QoL [24]; furthermore, as is known, the epiretinal membrane can cause visual disturbances and metamorphopsia, which impact the quality of vision regardless of visual acuity [8]. In this study, our analysis focused on symptomatic patients who were candidates for surgery, with included eyes classified as ≥Stage 2 and no eyes falling into Stage 1.

Several previous reports have employed Radner reading chart-derived metrics to quantify reading performance in eyes with macular pathologies, especially age-related macular degeneration, showing a significant association with BCVA [25,26,27,28]. If we look specifically at macular surgical pathology, Cappello et al. [15] investigated reading ability for macular hole and macular pucker, demonstrating again a trend of near vision in line with the BCVA in 12 months of follow-up, with an improvement in both parameters following surgery. In our study, BCVA, reading acuity, and maximum reading speed showed the same trend, in accordance with the literature, with significant worsening in the transition from Stage 3 to Stage 4. Moreover, univariate and multivariate analysis with BCVA set as the dependent variable identified both reading acuity and maximum reading speed as factors significantly associated with BCVA.

Although it is possible to speculate that near vision is more sensitive than far vision in the early detection of macular pathology, in our study, we did not detect a worsening of near vision prior to the worsening of BCVA as the stages progressed. In fact, both BCVA and the near vision parameters remained stable from Stage 2 to Stage 3. Few studies have considered this aspect in detail; a previous report found an improvement in near vision after surgical ERM removal even when BCVA did not improve [29]. Hoerster et al. [30] explored the sensitivity of reading performance in detecting subclinical changes during screening, with percentages of reduced reading ability in around 10% of neovascular AMD recurrences, similar to those obtained with the Amsler test.

Notably, in our study, we showed a significant worsening of the functional parameters of far and near vision only in the transition from Stage 3 to Stage 4, despite an overall worsening trend as the OCT stage progressed. The same result was obtained for the structural parameter of CMT, which increased significantly from Stage 3 to Stage 4.

A previous study evaluated functional and structural aspects in different stages of ERM, also including asymptomatic eyes [14]. This study employed the classification proposed by Mathews and colleagues [31], which divided the membranes into three stages according to the ratio between the thickness of the fovea and the parafovea, showing a significant difference in BCVA (in addition to FAZ area, FAZ perimeter, and superficial vessel density) between the three stages of ERM. Comparing the two OCT grading systems, Mathews grade 0 corresponded to Govetto Stage 1, Mathews grade 1 included both Govetto Stage 2 and 3, and grade 2 corresponded to Stage 4. Therefore, the functional difference identified in the transition from one stage to another corresponded to that detected in our study. Another previous study focusing on OCTA characteristics in different stages of ERM, starting from stage 1, reported among the secondary outcomes a significant overall worsening of the BCVA and an increase in CMT in accordance with the increase in ERM grading; however, the authors did not mention the statistical significance of the transition from one stage to another [32].

Given the results obtained, it is evident that the main structural and functional differences are inherent in the passage between Stages 3 and 4. As is known, the majority of ERMs remain stable over time, while the remaining ones progress or regress in an equal percentage [3], and today, surgical timing is still debated. It has been proven that the later the stage at which surgery is performed and the deeper the ERM-induced retinal changes are, the slower and more challenging the visual recovery of the patient after surgery is [23]. However, on the contrary, more recent evidence would seem to suggest that the deferral of surgery in eyes with progressing ERMs does not cause a disadvantage in final visual acuity compared with immediate surgery [24]. What we have highlighted might allow the surgeon to have an additional element for choice. In light of the above, if we focus on symptomatic membranes with documented progression, the choice of the surgeon should not underestimate the crucial role of the functional and structural boundary line existing between Stage 3 and Stage 4, while it would seem less important the line between Stage 2 and 3.

Our study has limitations which should be weighed up when interpreting our results. A main limitation of the study is its retrospective nature. Based on existing records, valuable clinical data such as the assessment of complete or incomplete posterior vitreous detachment (PVD) via slit lamp biomicroscopy were not available. Another limitation arises from the relatively small sample size of patients included. A larger cohort might have yielded significant distinctions even between Stages 2 and 3. Furthermore, the employed reading tests were monocularly administrated, which may inadequately represent binocular reading performance in daily activities. Also, patients with advanced ERMs can be characterized by fixation instability; thus, it remains possible that this factor partially confounded our analysis. Despite this, studies using Radner reading charts have shown a significant relationship with diseases that notably affect this aspect, like neovascular AMD and macular atrophy secondary to it, and good inter-session repeatability [26,27].

## 5. Conclusions

In conclusion, this study has demonstrated that in eyes affected by ERM and candidates for surgery, OCT staging has a significant functional and anatomical impact during the transition from Stage 3 to Stage 4. This transition is associated with a significant reduction in both distance and near vision, as well as a notable increase in CMT, changes not observed in earlier stages. Such findings may provide valuable insights for streamlining the current approach to membrane classification and offer additional guidance for surgical timing. However, further studies are warranted to validate the observed outcomes.

## Figures and Tables

**Table 1:** Demographics and clinical characteristics in different stages of idiopathic epiretinal membrane.

	Stage 2 (n = 10)	Stage 3 (n = 24)	Stage 4 (n = 10)	*p*-Value *
Age, years (±SD)	73.40 (±4.08)	74.09 (±3.33)	69.25 (±6.75)	0.225 ^a^
Gender				
Women, n (%)	4 (40.0%)	10 (41.7%)	5 (50.0%)	0.881 ^b^
Men, n (%)	6 (60.0%)	14 (58.3%)	5 (50.0%)	
Lens Status				
Pseudophakic, n (%)	4 (40.0%)	11 (45.8%)	4 (40.0%)	0.927 ^b^
Phakic, n (%)	6 (60.0%)	13 (54.2%)	6 (60.0%)	
Axial length, mm (±SD)	24.14 (±1.08)	24.35 (±1.21)	24.22 (±1.33)	0.753 ^b^

* *p*-values ≤ 0.05 were considered significant. SD: standard deviation. ^a^ Kruskal–Wallis test. ^b^ Pearson chi-squared test.

**Table 2:** Functional parameters in different stages of idiopathic epiretinal membrane.

	Stage 2 (n = 10)	Stage 3 (n= 24)	Stage 4 (n = 10)	*p*-Value *
BCVA, LogMAR (±SD)	0.31 (±0.16)	0.38 (±0.20)	0.71 (±0.24)	**0.008** ^a^
*p*-value (Mann–Whitney)	0.461 ^b^	**0.012** ^c^	**0.003** ^d^	
Reading acuity, LogRAD (±SD)	0.61 (±0.20)	0.64 (±0.16)	0.84 (±0.06)	**0.003** ^a^
*p*-value (Mann–Whitney)	0.702 ^b^	**0.002** ^c^	**0.004** ^d^	
Maximum RS, wpm (±SD)	125.63 (±57.79)	109.10 (±50.57)	47.29 (±30.06)	**0.006** ^a^
*p*-value (Mann–Whitney)	0.473 ^b^	**0.013** ^c^	**0.003** ^d^	
Amsler grid test, n (%)	6 (60.0%)	18 (75.0%)	10 (100.0%)	0.095 ^e^

* *p*-values ≤ 0.05 were considered significant. BCVA: best-corrected distance visual acuity (LogMAR (logarithm of the minimum angle of resolution)); SD: standard deviation; LogRAD: logarithm of reading acuity determination; RS: reading speed (wpm (words per minute)). ^a^ Kruskal–Wallis test. ^b^ Versus Stage 3 at each location. ^c^ Versus Stage 4 at each location. ^d^ Versus Stage 2 at each location. ^e^ Pearson chi-squared test.

**Table 3:** OCT structural parameters in different stages of idiopathic epiretinal membrane.

	Stage 2 (n = 10)	Stage 3 (n= 24)	Stage 4 (n = 10)	*p*-Value *
CMT, μm (±SD)	444.50 (±49.02)	485.22 ± 46.90	618.00 ± 109.93	**0.004** ^a^
*p*-value (Mann–Whitney)	0.191 ^b^	**0.006** ^c^	**0.003** ^d^	
Cotton ball sign, n (%)	3 (30)	2 (8.3)	0 (0)	-
Foveolar detachment, n (%)	1 (10)	1 (4.2)	0 (0)	-
Acquired vitelliform lesion, n (%)	1 (10)	3 (12.5)	0 (0)	-
EZ disruption, n (%)	1 (10)	4 (16.7)	4 (40)	0.292 ^e^
Tractional cysts, n (%)	3 (30)	12 (50)	4 (40)	0.548 ^e^

* *p*-values ≤ 0.05 were considered significant. CMT: central macular thickness; EZ: ellipsoid zone. ^a^ Kruskal–Wallis test. ^b^ Versus Stage 3 at each location. ^c^ Versus Stage 4 at each location. ^d^ Versus Stage 2 at each location. ^e^ Pearson chi-squared test.

**Table 4:** OCT angiography parameters in different stages of idiopathic epiretinal membrane.

	Stage 2 (n = 10)	Stage 3 (n= 24)	Stage 4 (n = 10)	*p*-Value *^,a^
FAZ parameters				
Area, mm^2^ (±SD)	0.073 ± 0.058	0.069 ± 0.051	0.058 ± 0.041	0.885
Perimeter, mm (±SD)	1.29 ± 0.65	1.21 ± 0.45	1.14 ± 0.43	0.915
Superficial capillary plexus				
Central VD, % (±SD)	14.63 ± 4.78	15.14 ± 4.94	15.24 ± 1.98	0.673
Inner VD, % (±SD)	18.01 ± 4.51	18.27 ± 3.21	19.53 ± 2.48	0.662
Total VD, % (±SD)	17.42 ± 4.31	17.90 ± 3.31	18.18 ± 2.32	0.999
Central perfusion, % (±SD)	26.88 ± 9.12	27.49 ± 8.74	29.68 ± 3.94	0.957
Inner perfusion, % (±SD)	34.13 ± 7.35	35.60 ± 5.34	37.68 ± 5.87	0.336
Total perfusion, % (±SD)	33.51 ± 7.68	34.68 ± 5.61	37.68 ± 3.37	0.255

* *p*-values ≤ 0.05 were considered significant. FAZ: foveal avascular zone; VD: vessel density. ^a^ Kruskal–Wallis test.

**Table 5:** Multivariate logistic regression with BCVA set as a dependent variable.

Independent Variables	Exp(B)	CI 95%	*p*-Value
Reading acuity	0.010	0.001–0.018	**<0.01**
Maximum RS	0.011	0.006–0.016	**<0.01**
ERM stage	0.034	0.012-0.108	0.361

BCVA: best-corrected visual acuity; CI: confidence interval; RS: reading speed; ERM: epiretinal membrane.

## Data Availability

The data that support the findings of this study are available from the corresponding author upon reasonable request.
